# Guanine-Rich Sequences Are a Dominant Feature of Exosomal microRNAs across the Mammalian Species and Cell Types

**DOI:** 10.1371/journal.pone.0154134

**Published:** 2016-04-21

**Authors:** Fumiyasu Momose, Naohiro Seo, Yasushi Akahori, Shin-ichi Sawada, Naozumi Harada, Toru Ogura, Kazunari Akiyoshi, Hiroshi Shiku

**Affiliations:** 1 Department of Immuno-Gene Therapy, Mie University Graduate School of Medicine, Edobashi, Tsu, Mie 514–8507, Japan; 2 Graduate School of Engineering, Kyoto University, Katsura, Nishikyo-ku, Kyoto 615–8510, Japan; 3 ERATO Bio-Nanotransporter Project, Japan Science and Technology Agency (JST), Tokyo 102–0076, Japan; 4 Clinical Research Support Center, Mie University Hospital, Tsu Mie 514–8507, Japan; University of Bristol, UNITED KINGDOM

## Abstract

Exosome is an extracellular vesicle released from multivesicular endosomes and contains micro (mi) RNAs and functional proteins derived from the donor cells. Exosomal miRNAs act as an effector during communication with appropriate recipient cells, this can aid in the utilization of the exosomes in a drug delivery system for various disorders including malignancies. Differences in the miRNA distribution pattern between exosomes and donor cells indicate the active translocation of miRNAs into the exosome cargos in a miRNA sequence-dependent manner, although the molecular mechanism is little known. In this study, we statistically analyzed the miRNA microarray data and revealed that the guanine (G)-rich sequence is a dominant feature of exosome-dominant miRNAs, across the mammalian species-specificity and the cell types. Our results provide important information regarding the potential use of exosome cargos to develop miRNA-based drugs for the treatment of human diseases.

## Introduction

Exosomes are extracellular vesicles, ranging in size from 40 to 150 nm in diameter, which are released from variety cell types by the exocytotic fusion of multivesicular bodies of the endosome with the plasma membrane [1]. Proteins and lipids are the major components of exosome membranes. Proteins on the exosome membranes are thought to function as ligands for interactions with target cells. In addition, various nucleic acids, including mRNAs and microRNAs (miRNAs), are found in the exosomal lumen [1,2]. Evidence suggests that miRNAs in exosome cargos are able to modulate appropriate neighboring cells or distant recipient cells [1–3], however the exact molecular mechanisms of endocytosis and the specific interaction between exosomes and target cells is yet to be clarified.

miRNAs are small (17–24 ribonucleic acids), non-coding RNAs (located mainly within genomic introns) that regulate post-transcriptional gene silencing by binding to the 3’-untranslated region (UTR) or open reading frame of target mRNAs [4]. It has been reported that miRNAs are present in body fluids including blood [5], urine [6], breast milk [7], saliva [5] and cerebrospinal fluid [8] by loading of high-density lipoproteins [9] or binding of argonaute (Ago) 2 and GW184 proteins [10,11] without packaging with exosome cargos. However, vesicle-free miRNAs are difficult to deliver to distant recipient cells due to reduced specificity and instability. Conversely, several studies have reported that exosomal miRNAs participate in cell-to-cell communication in pathological conditions (such as immune responses to tumor progression and regression) via apoptotic death, migration, growth, and differentiation of target cells [1–3]. Taken together, exosome cargos have a crucial role in RNA stabilization and interacting with target cells.

Difficulties in introducing miRNAs and small interfering RNAs (siRNAs) directly into the exosome lumen by physical manipulations, including electroporation, have been reported. It seems that the nano-size membrane vesicles do not form enough holes to allow miRNAs to enter. Also, the exosome often aggregates with the precipitated small RNAs after electroporation [12]. Introduction of the certain miRNAs into exosomes after transfection with electroporation or lipofection of synthetic miRNAs or miRNA-expressing vectors into the parent cells seems to obtain feasible results in some cases [13, 14], However, no such success has been achieved using siRNAs, which are designed at lower guanine (G)/cytosine (C) values [15], implying that the translocation of miRNAs from a parent cell into the exosome cargo is dependent on the G/C content of the miRNA sequences. It has recently been reported that exosomal miRNAs are concentrated according to specific motifs, including GGAG, UGAG, CCCU and UCCU, in a sumoylated RNA-binding protein (RBP: RNPA2B1)-mediated manner [16], while some exosome-dominant miRNAs are out of this rule.

In this study, we performed statistical sequence analysis of miRNAs in exosomes from various kinds of cells including human T cells, human tumor cells, murine T cells, and murine macrophage cells, and found ubiquitous G-concentrated sequences, but not 4-base motifs, and predicted RBPs specific for these G-rich exosomal miRNAs. Our study will be useful for the production of functional miRNA-enriched exosomes that could be used in drug delivery systems.

## Materials and Methods

### Mice

Female BALB/c mice aged 6–8 weeks were obtained from SLC Japan. H-2K^d^-restricted and mutated (m) ERK2 136–144 peptide-specific TCR (Vα10.1/Jα48 and Vβ8.3/Dβ2.1/Jβ2.6) gene-transfected DUC18 mice [17] and BALB/c mice were maintained at the Experimental Animal Facility of Mie University, and used at 8-10-weeks old. The Ethics Review Committee for Animal Experimentation of Mie University approved the experimental protocols (Approval No.: 23–8).

### Cultivation of human PBMCs, human tumor cells, murine T cells, and murine macrophages

Peripheral blood mononuclear cells (PBMCs) were isolated from the whole blood of healthy donors by Ficoll-density gradient separation, and cultured for two weeks at a concentration of 2 x 10^5^ cells/ml in GT-T503 medium (Takara Bio) supplemented with 0.6% autologous plasma, 0.2% human serum albumin (HSA; CSL-Behring), and 600 IU/ml recombinant (r) human IL-2 in 5 μg/ml anti-human CD3 mAb (OKT-3: eBioscience)- and 25 μg/ml RetroNectin (Takara Bio)-coated plates. The study was conducted in accordance with the current version of the Declaration of Helsinki. Written informed consent was obtained from all healthy donors participating in this study. The experimental protocols were approved by the Ethics Review Committees of the Mie University Graduate School of Medicine (Approval No.: 2879).

Murine CTLs were obtained from splenocytes of DUC18 mice. DUC18 splenocytes were cultured for 3 days at a concentration of 0.5 x 10^6^ cells/ml in RPMI-1640 medium (Sigma-Aldrich) containing 1 μg/ml of mERK2 peptide (QYIHSANVL) and 10% exosome-depleted FCS, and then supplemented with 100 IU/ml rIL-2 for a further 4 days. Murine T cells were obtained by the cultivation of BALB/c CD8^+^ splenocytes and subcutaneous CMS5a (fibrosarcoma cell line: [17])-bearing BALB/c splenocytes. Hemolyzed splenocytes were cultured for 3 days at a concentration of 0.5 x 10^6^ cells/ml in RPMI-1640 medium (Sigma-Aldrich) supplemented with 10 IU/ml rIL-2, 1 μg/ml anti-murine CD28 mAb (37.51: eBioscience) and 10% exosome-depleted FCS in anti-murine CD3 mAb (145-2C11: eBioscience)-immobilized 6-well plates, and then supplemented with 100 IU/ml rIL-2 for a further 4 days.

A549 cells (human lung carcinoma cell line), HCT116 cells (human colorectal carcinoma cell line), K562 cells (human leukemia cell line) and RAW264.7 cells (murine macrophage-like cell line) (All from ATCC without any restrictions) were cultured in D-MEM (A549 and HCT116) or RPMI-1640 (K562 and RAW264.7) medium (Wako Pure Chemical Industries) supplemented with 10% exosome-depleted FCS. Culture supernatants were collected after 1 week and used for exosome purification. Exosome-depleted FCS was prepared by filtration through 0.45 μm and 0.22 μm filters (Merck Millipore) after ultracentrifugation at 100,000 *g* for 6 hrs.

### Isolation and purification of exosomes

**Fig 1:**
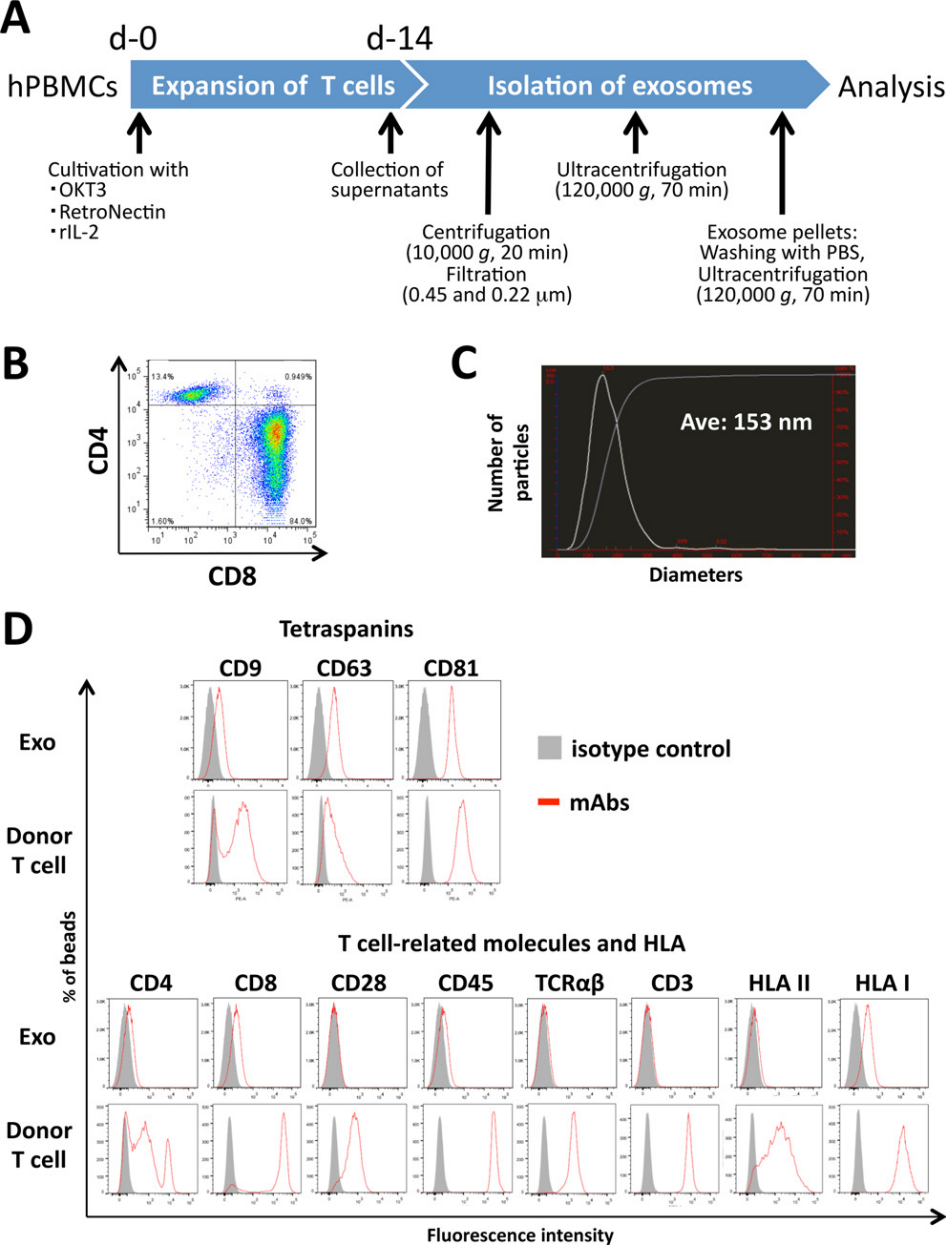
Characterization of activated human T cell-released exosomes. (A) Study design for the preparation of activated human T cell-released exosomes. Day 14 culture supernatant of OKT3-, RetroNectin- and rIL-2-activated human PBMCs were collected and subjected to ultracentrifugation after removing cell debris and protein aggregations. (B) Day 14 OKT3-, RetroNectin- and rIL-2-activated human PBMCs were examined for CD4 and CD8 expression using flow cytometry. (C) Mean diameter of the activated human T cell-derived exosomes, measured by NanoSight LM10. (D) Expression of tetraspanins (CD9, CD63, and CD81), T cell markers (CD3, CD4, CD8, CD28, CD45, and pan-TCRαβ) and HLAs (pan-HLA-I and pan-HLA-II) of the day-14 activated human T cells (Donor T cell) or their released exosome bound with latex beads (Exo) were determined by flow cytometry after staining with molecule-specific monoclonal antibodies or isotype controls.

Exosomes were prepared from culture supernatants by differential centrifugation and filtration. Cell cultures were subjected to centrifugation at 400 *g* for 10 min and 10,000 *g* for 20 min to remove cells and debris, and then filtration through 0.45 μm and 0.22 μm filters (Merck Millipore). Exosomes were purified from the culture supernatants by ultracentrifugation (Optima L-90K, Beckman Coulter; SW28 rotor) at 120,000 *g* for 70 min at 4°C, and then washed with phosphate-buffered saline (PBS). Finally, the exosome pellets were dissolved in PBS at a maximum concentration of 300 μg/ml, and stored at either 4°C or -80°C (Fig 1A).

### Protein assays

Total exosomal protein was measured by a bicinchoninic acid (BCA) assay (Micro BCA Protein Assay kit, Thermo Scientific) and analyzed using a microplate reader and SoftMax Pro Software (SpectraMax M2, Molecular Devices).

### Measuring number and size of exosomes

The number and size of exosomes were determined by nanoparticle-tracking analysis (NTA) based on Brownian motion of nanovesicles using a NanoSight LM10 microscope and NTA software ver. 2.3 (NanoSight).

### Flow cytometric analysis of exosome surface proteins

Latex beads were used to detect proteins on the surface of exosomes by flow cytometry. Ninety microliters of aldehyde/sulfate latex beads solution (4 μm diameter; A37304: Life Technologies) were washed 3 times with 0.025 M 2-(N-morpholino) ethanesulfonic acid (MES) buffer, and adjusted in 180 μl of MES. Per sample of flow cytometric analysis, 2 μl of beads solution was mixed with 6 μg exosome/PBS solution, and adjusted in 1 ml of MES. After incubation at room temperature for 2 h with a rotator, 300 μl of 400 mM glycine was added and further incubated for 30 min to block the surface of the latex beads. Exosome-coated beads were washed three times with 2% exosome-depleted FCS/PBS and then stained with PE-conjugated anti-human CD9 (eBioSN4: eBioscience), anti-human CD63 (MEM-259: Beckman Coulter), anti-human CD81 (JS-81: BD Biosciences), anti-human HLA-ABC (W6/32: eBioscience), anti-mouse CD63 (NVG-2: Biolegend), anti-mouse CD81 (Eat-2: Biolegend) mouse IgG1κ (P3.6.2.8.1: eBioscience), mouse IgG2a (eBM2a: eBioscience) or rat IgG2a (eBR2a: eBioscience) mAb, or FITC-conjugated anti-human CD3 (HIT3a: BD Biosciences), anti-human CD4 (RPA-T4: BD Biosciences), anti-human CD8 (HIT8a: BD Biosciences), anti-human CD45 (HI30: eBioscience), anti-human TCRαβ (IP26: eBioscience), anti-mouse CD8 (53.67: BD Biosciences), anti-mouse CD9 (DRAP-27: Biolegend), anti-mouse H-2K^d^ (SF1-1.1.1: eBioscience), mouse IgG1κ (P3.6.2.8.1: eBioscience), or rat IgG2a (RTK2758: Biolegend) mAb at 4°C for 20 min. The stained exosome-coated beads were washed twice with 0.5% FBS/PBS and subjected to flow cytometry (FACSCanto, Becton Dickinson). The data were further analyzed using Flowjo software (Tomy Digital Biology).

### RNA isolation and miRNA microarray analysis

Small RNAs were extracted from the exosome or cell samples by using 3D-gene extract reagent (Toray) or miRNeasy mini kit (Qiagen), respectively, according to a manufacturer’s instruction. The extracted RNAs were checked by Bioanalyzer (Agilent) and was labeled with 3D-Gene miRNA labeling kit (Toray), and hybridized onto 3D-Gene Human miRNA Oligo chips (Platform GPL18941: Toray). The annotations and oligonucleotide sequences of the probes were conformed to the miRBase (http://www.mirbase.org/). After stringent washes, fluorescent signals were scanned with the 3D-Gene Scanner and analyzed using 3D-Gene extraction software (Toray).

The raw data of each spot was normalized by substitution with a mean intensity of the background signal determined by all blank spots’ signal intensities of 95% confidence intervals. Measurements of spots with the signal intensities greater than 2 standard deviations (SD) of the background signal intensity were considered to be valid. A relative expression level of a given miRNA was calculated by comparing the signal intensities of the valid spots throughout the microarray experiments. The normalized data were globally normalized per array, such that the median of the signal intensity was adjusted to 25.

### Reverse transcription quantitative PCR (RT-qPCR)

Small RNA was isolated from exosomes or donor cells using miRNeasy mini kit (Qiagen) according to the manufacture’s direction. Reverse transcription of RNA was performed using the Mir-X miRNA First-Strand Synthesis Kit (Clonetech). RT-qPCR was performed by StepOnePlus Real-Time PCR system (Applied Biosystems) using SYBR Advantage qPCR Premix (Clonetech) and synthetic primers (GeneDesign). Quantity of each miRNA was measured by the comparative Ct method (the ^⊿⊿^Ct method). The level of each miRNAs was normalized to that of a U6 snRNA control.

### Analysis of miRNA microarray data and sequences

Human activated T cells or their-released exosomes were prepared from PBMCs of 3 healthy donors including 2 lots from one donor. Exosome- or donor human T cell-dominant miRNAs were selected that showed over a 2-fold change in the comparison between the two, and then visualized as a heat map of the cluster analysis. The normalized data were Z-transformed and a heat-map of selected genes was constructed by hierarchical cluster analysis using Cluster 3.0 software and the results were displayed with the TreeView program (http://rana.lbl.gov/eisen/).

Total numbers of 256 (4^4^) patterns of 4 nucleotide motifs in 157 exosome (E/C value > 8)—and 76 donor cell (C/E value > 2) -dominant miRNA sequences common among donors were counted using Excel software, and 29 significantly high patterns (exosome-dominant p < 0.05; donor cell-dominant: p < 0.001) of 4 nucleotide motifs were extracted by Fisher’s exact test.

### Analysis of ratio and pattern of guanine in miRNA sequences

To forecast of G-rich character of exosomal miRNAs, miRNA sequences from human T cell (the averaged raw data from the miRNA microarray analysis of three donors)-, K562- DUC18 CTL-, or RAW264.7-released exosomes containing G motifs (G, GG, GGG, GGGG) as red letters and other bases (A, U, C) as white letters were lined up in order from the highest value of exosomal miRNA or ratio of exosomal/donor T cell miRNA by using Excel VBA macro programing. The percentage of G, the maximal continuity of G, the number of continuous G, and the maximal G-G interval of miRNA sequences from human T cell-, A549-, or HCT116-released exosomes were counted and analyzed using Excel software before statistical analysis. The percentage of each base in exosomal miRNAs and donor T cell miRNAs was indicated by different colors, and lined up in order from the highest value of exosomal miRNAs or donor T cell miRNAs, or the highest exosomal/donor T cell miRNA ratio. In addition, the percentage of each base in miRNAs from A549-, HCT116-, K562-, BALB/c CD8^+^ T-, DUC18 CTL-, or RAW264.7-released exosomes was indicated by different colors and lined up in order from the highest value of exosomal miRNAs for estimate G-rich features of exosomal miRNA across the cell types.

### Statistical analysis

Pearson’s correlation test was performed to confirm statistical significance of a G-rich feature of exosome-dominant miRNA sequences. The correlation coefficient (r) and p-value were calculated between the percentage of G, the maximal continuity of G, the number of continuous G, or the maximum G-G interval and the exosome/donor cell miRNA ratios, and between the percentage of each base and the amount of the miRNA (normalized raw data from the miRNA microarray) or the exosome/donor cell miRNA ratios. Positive or negative correlation was evaluated as “significant” at a correlation coefficient 0 < r ≦ 1 or -1 ≦ r < 0, respectively, and p < 0.05.

## Results

### Isolation and characterization of activated human T cell-, human tumor cell-, murine T cell- and murine macrophage-released exosomes

hPBMCs were cultured for two weeks in medium supplemented with OKT3, RetroNectin, rIL-2 and autologous plasma, and the proportion of CD4^+^ or CD8^+^ cells was determined by flow cytometry. This protocol always resulted in a dominant expansion of the CD8^+^ cell population (Fig 1A and 1B). The supernatant obtained from these cultured hPBMCs was subjected to ultracentrifugation after removing cell debris and aggregated proteins for exosome purification (Fig 1A). The hPBMC-released exosomes exhibited an average diameter of 153 nm, as determined by NTA (Fig 1C). In addition to the dominancy of CD8^+^ cell population, nevertheless the 2-weeks cultured hPBMCs abundantly expressed tetraspanins (CD9, CD63, and CD81), T cell-related molecules (CD3, CD28, CD45, T cell receptor [TCR] αβ), and human leukocyte antigens (HLA class I and class II molecules), their-released exosomes exhibited the modest expression of CD4, CD8, CD9, CD45, and HLA class II molecules and the substantial expression of CD63, CD81, and HLA class I molecules by flow cytometric analysis of the mAb-stained exosome/latex beads, while no expression of CD3, CD28, or TCRαβ was detected on the surface of the cultured human T cell-released exosomes (Fig 1D). Human tumor cell (K562, A549, and HCT116)-released exosomes showed vigorous expression of tetraspanins (CD9, CD63, and CD81) with the exception of CD9 on K562 exosomes at approx. 150 nm diameter. HLA expression was observed on only HCT116-released exosomes (S1 Fig). On the other hand, although CD9, CD63, CD81, and H-2K^d^ were sufficiently expressed on the murine RAW264.7-released exosomes, DUC18 CTL- or BALB CD8^+^ T-released exosomes did not exhibit CD63 or CD81 expression in addition to the weak expression of CD9 or H-2K^d^. Unexpectedly, no expression of CD8 was shown on DUC18 CTL or BALB CD8^+^ T cell-released exosomes (S2 Fig).

### Difference in the miRNA distribution between exosomes and donor cells

**Fig 2:**
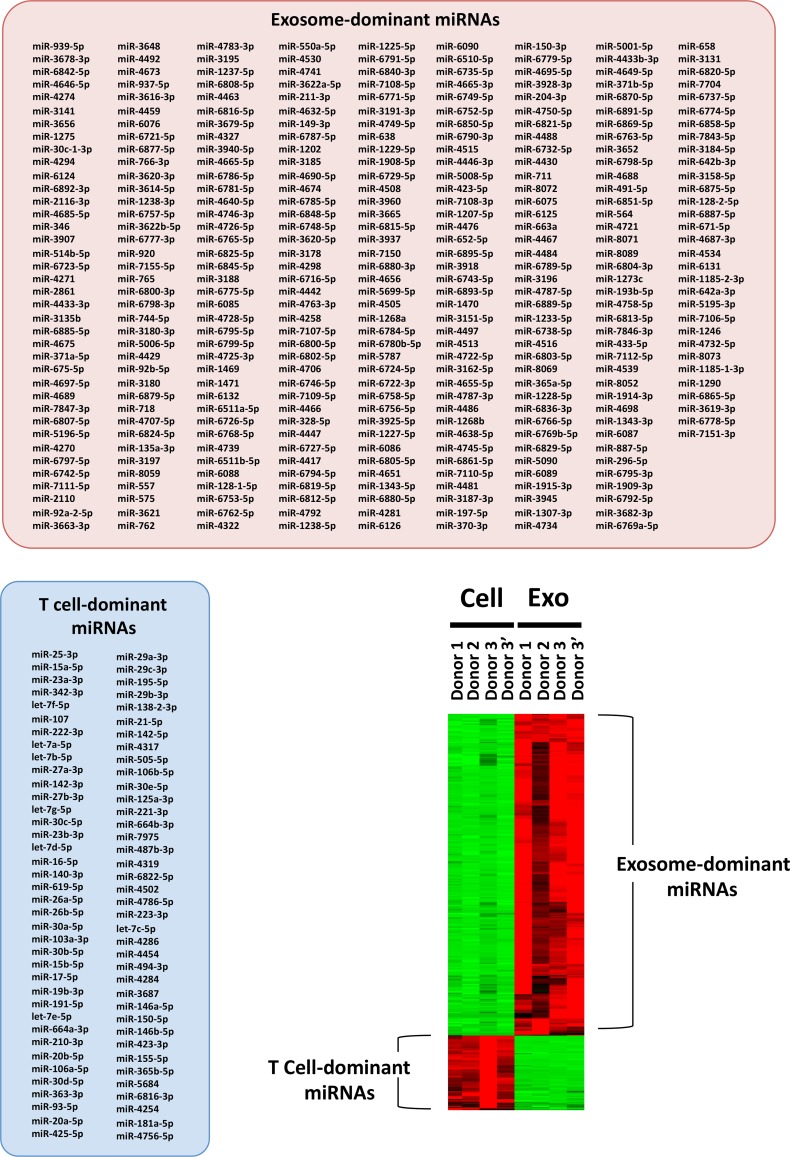
Difference in the distribution between exosome-dominant miRNAs and donor T cell-dominant miRNAs. Three hundred and thirty five exosome (E/C > 2)- and 76 donor human T cell (C/E > 2)-dominant miRNAs were selected by reciprocal comparison of the values of the normalized raw data of miRNA microarray. In addition, differences in the amounts of each exosome- and donor T cell-dominant miRNA were visualized as a heat map, in the red column.

Human activated T cells and their released exosomes were prepared from the PBMCs of three healthy donors, including two lots from one donor, and were subjected to miRNA microarray analysis. A comparative analysis of exosomal miRNAs and the corresponding donor cell-derived miRNAs was performed. As shown in Fig 2, we selected 335 exosome-dominant miRNAs (E/C > 2) and 76 donor T cell-dominant miRNAs (C/E > 2), and visualized the data as a heat map. Normalized raw data of miRNA microarray of representative miRNAs (6 exosome-dominant miRNAs with high values or E/C ratios, or 3 donor cell-dominant miRNAs with high values) were confirmed to correlate with the values of relative quantification using RT-qPCR (S3 Fig). These results indicate differences between the composition of the exosomal miRNAs and the donor T cell-derived miRNAs.

### Prediction of G-rich feature of exosomal miRNAs

**Fig 3:**
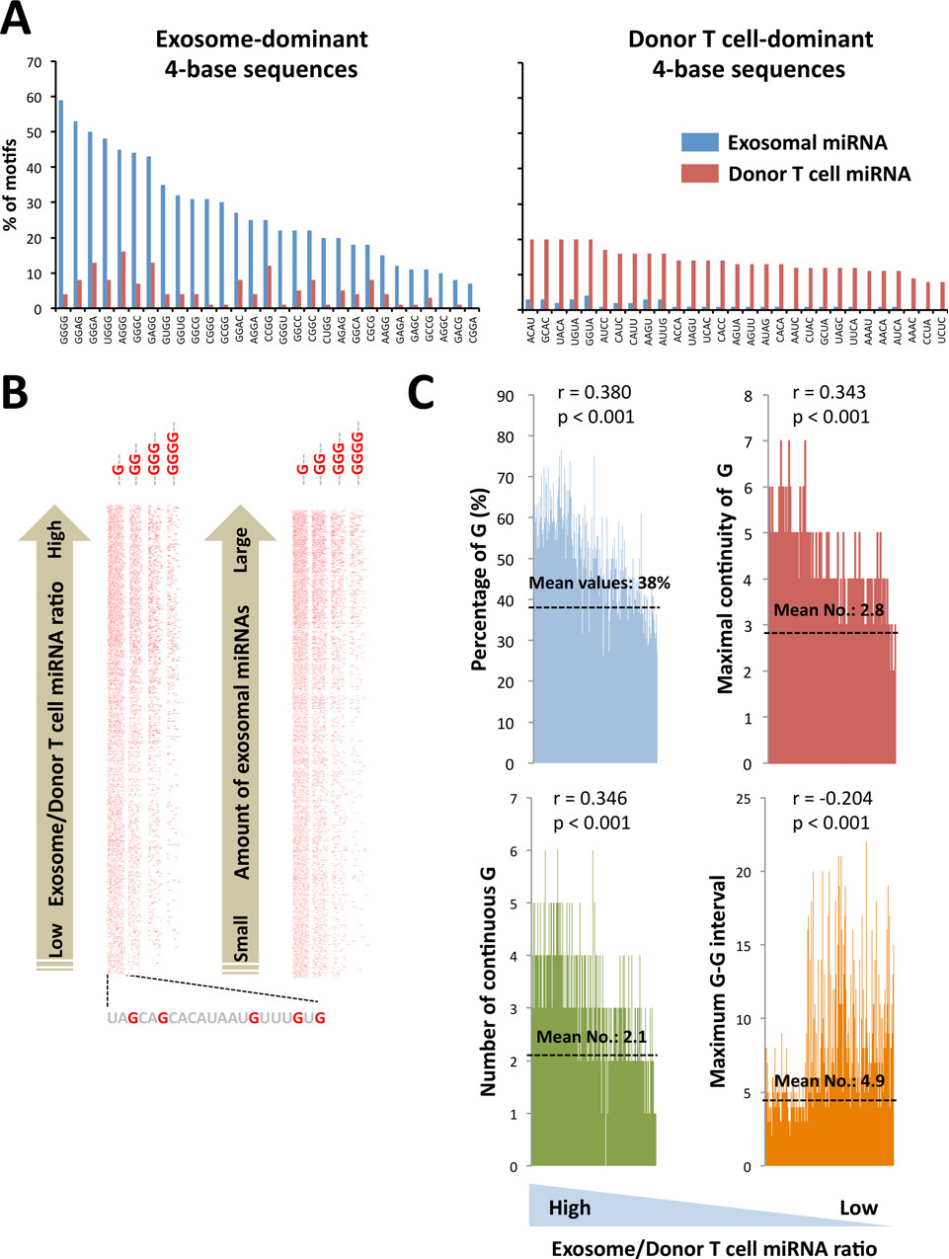
Enrichment of G in human T cell-released exosome-dominant miRNA sequences. (A) Total content numbers of 256 patterns of 4 nucleotides were examined in 157 exosome (E/C > 8)- and 76 donor human T cell (C/E > 2)-dominant miRNA sequences. Exosome-dominant miRNAs have a G-rich feature, such as GGGG, GGGA, AGGG and GAGG, as determined by Fisher’s exact test. There was no specific pattern in the donor T cell-dominant miRNA sequences. (B) The indicated G patterns (no color in other bases and patterns) were visualized in miRNA sequences as a red color, and lined up in order from the highest exosome/donor T cell miRNA ratio or the largest amount of exosomal miRNAs. (C) Percentage of G, maximal continuity of G, number of continuous G and maximum G-G interval in 838 miRNA sequences were lined up in order from the highest ratio of exosome/donor T cell miRNAs. Pearson’s correlation test was performed, and the correlation coefficient (r) and p-value were calculated to confirm statistical significance of each G feature of exosome-dominant miRNA sequences.

A total of 256 patterns of 4 nucleotide sequences in 157 exosome- and 76 donor T cell-dominant miRNA sequences were examined. Human CD8^+^ T cell-derived exosome-dominant miRNA sequences included G-rich sequences, such as GGGG, GGGA, AGGG and GAGG at a high frequency, which is similar with one motif from other group [16], whereas there was no such specific feature in the parent cell-dominant miRNA sequences (Fig 3A). This G-enrichment was visualized by lining each miRNA sequence up in order from the highest exosome/donor T cell miRNA ratio or the highest amount of normalized raw data of exosomal miRNAs (Fig 3B). Statistical analysis of the percentage of G (r = 0.380, p < 0.001), the maximal continuity of G (r = 0.343, p < 0.001), the number of continuous G (r = 0.346, p < 0.001), and the maximum G-G interval (r = -0.204, p < 0.001), exhibited the concentrated G in exosomal miRNA sequences (Fig 3C). Consistent with the human T cell-released exosome-dominant miRNA sequences, the concentrated G regions also seemed to be present in the miRNA sequences of murine cytotoxic T lymphocyte (CTL)-, human K562 cell-, or murine RAW264.7 cell-derived exosomes (S4 Fig). In addition, the enrichment of G was shown statistically significance in A549- and HCT116-released exosome-dominant miRNAs (S5 Fig) similar to the miRNAs in human T cell-released exosomes, indicating that the conserved G-rich phenomenon of exosomal miRNAs is not restricted to one species or cell type.

### Statistical analysis to confirm the G abundance in human T cell-released exosome-dominant miRNA sequences

**Fig 4:**
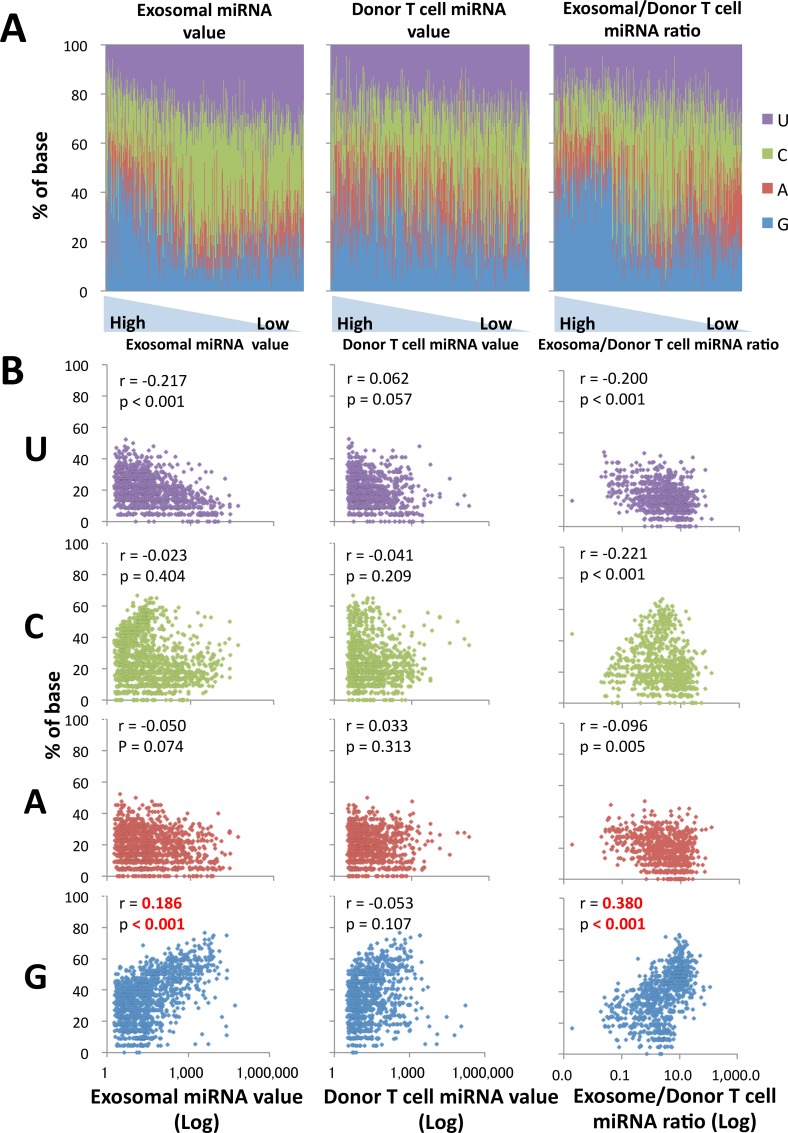
Statistical anaylsis of each 4 base in human T cell-released exosome-dominant miRNA sequences. (A) The percentage of each base in exosomal miRNAs and donor T cell miRNAs is indicated by different colors, and lined up in order from the highest value of exosomal miRNAs or donor T cell miRNAs, or the highest exosomal/donor T cell miRNA ratio. (B) Pearson’s correlation test was performed to confirm statistical significance of the G-rich feature of human T cell-released exosome-dominant miRNA sequences. The correlation coefficient (r) and p-value were calculated between the percentage of each base (U, C, A or G) and the exosomal miRNA values, donor T cell miRNA values, or exosome/donor T cell miRNA ratios.

The percentage of each base was visualized using different colors, and then lined up in order of the highest value of exosomal miRNAs or donor T cell miRNAs, or the highest ratio of exosome/donor T cell miRNAs (Fig 4A). In addition to the high G content in exosome-dominant miRNA sequences, an inverse correlation of U and C content in miRNA sequences was seen in the exosomal miRNA and the exosomal/donor T cell miRNA ratio groups, but not in a donor T cell miRNA value group. To confirm the positive correlation of G and the negative correlation of U and C, we performed a statistical analysis of each base using Pearson’s correlation test. As indicated in Fig 4B, the significant positive or negative correlation between the base percentage and miRNA value or ratio was shown in G or U, respectively, in the groups of exosomal miRNA value and exosomal/donor T cell miRNA ratio, but not a group of donor T cell miRNA value. Cytosine negatively correlated only in an exosomal/donor T cell miRNA ratio group.

### Conserved G-rich sequence of exosome-dominant miRNAs across the mammalian species and donor cell types

**Fig 5:**
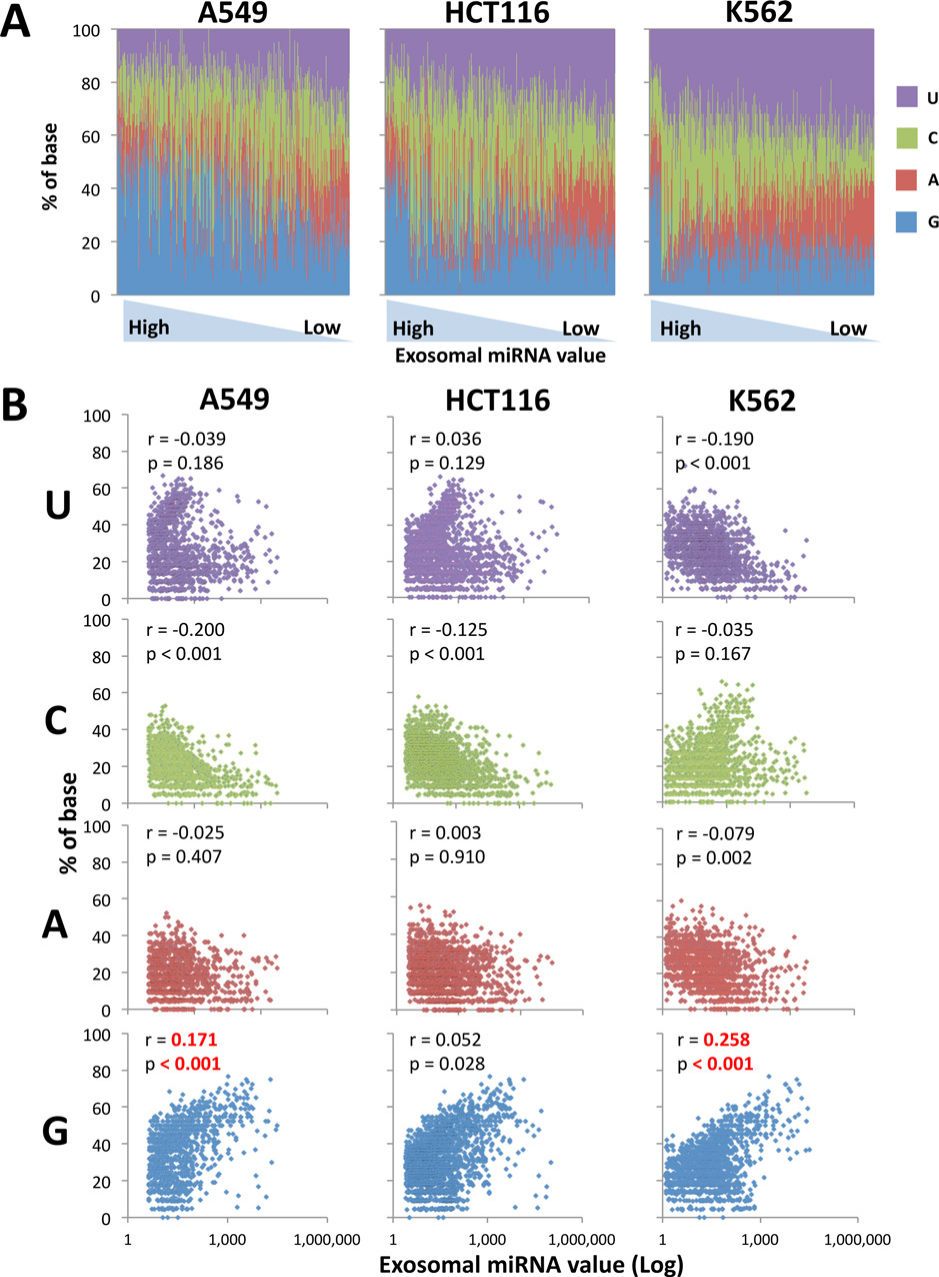
Statistical anaylsis of each 4 base in human tumor cell-released exosome-dominant miRNA sequences. (A) The percentage of each base in human tumor cell- (A549, HCT116 or K562) released exosome miRNAs is indicated by different colors, and are lined up in order from the highest value of exosomal miRNAs. (B) Pearson’s correlation test was performed to confirm statistical significance of the G-rich feature of human tumor cell-released exosomal miRNA sequences. The correlation coefficient (r) and p-value were calculated between the percentage of each base (U, C, A or G) and exosomal miRNA values.

**Fig 6:**
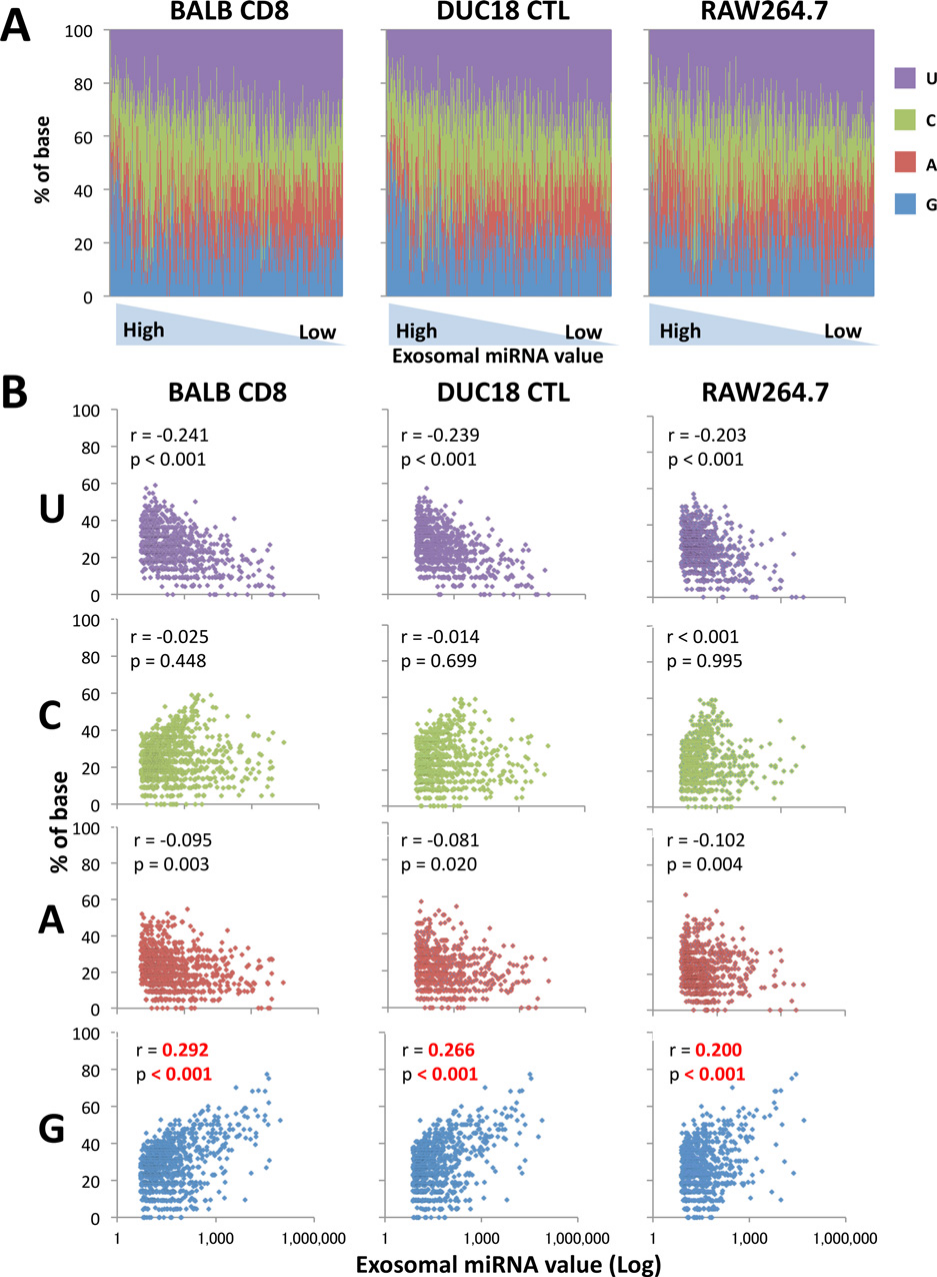
Statistical anaylsis of each 4 base in murine T cell-, CTL- and macrophage-released exosome-dominant miRNA sequences. (A) The percentage of each base in murine cell- (CD8^+^ T cells from BALB/c, DUC18 CTLs, or RAW264.7) released exosomal miRNAs is indicated by different colors and are lined up in order from the highest value of exosomal miRNAs. (B) Pearson’s correlation test was performed to confirm statistical significance of the G-rich feature of each murine cell-released exosomal miRNA sequence. The correlation coefficient (r) and p-value were calculated between the percentage of each base (U, C, A or G) and exosomal miRNA values.

Our data predict that the abundance of G in exosome-dominant miRNAs is conserved across the mammalian species and cell type (S4 and S5 Figs). Therefore, in exosomes from human tumor cells (A549, HCT116, and K562), murine T cells (BALB/c and CMS5a tumor-bearing BALB/c splenocytes), murine CTLs (mERK2 peptide-specific DUC18 splenocytes) and murine macrophages (RAW264.7), the G-rich features were evaluated by Pearson’s correlation test between percentage of each base and exosomal miRNA values (Figs 5A, 5B, 6A and 6B and S6 Fig) consistent with Fig 4. Exosomal miRNAs from human tumor cells, murine T cells, murine CTLs and murine macrophages exhibited a significantly positive correlation for G, while the G abundance in miRNA sequences from HCT116-released exosomes was little weak (Figs 5 and 6). On the other hand, there was a significant negative correlation of U and A in miRNA sequences from all types of murine cell-released exosomes, compared with the ambiguity of miRNA sequences form human tumor cell-derived exosomes (Figs 5B and 6B). Thus, we conclude that the G-rich sequence is a feature of mammal cell-released exosome-dominant miRNAs.

## Discussion

The functional strand of the mature miRNA, called as the guide strand, interacts with the Ago proteins by A- or U-recognition in 5’ region to form of the RNA-induced silencing complex with the target mRNA [18]. In addition, the seed sequence positioned at 5’ end of miRNA (nucleotide two to eight) plays a pivotal role in binding with 3’-UTR of mRNA and consequent translational repression and degradation of mRNA [19]. It has been reported that the G/C content in the seed sequence of miRNA exhibits positive correlation with specific recognition of target mRNA [20], indicating the profound significance of the concentrated G in exosomal miRNAs as demonstrated in this study by statistical analysis using Pearson’s correlation test. G-rich rule of exosomal miRNAs has a possibility to utilize exosome cargo in nucleotide acid medicine. For instance, transfection of the G-concentrated miRNA-expressing vectors into appropriate cells may result in the production of transfected miRNA-embedded exosomes.

Dendritic cell (DC) -derived exosomes directly modulate the antigen-specific responses of CD4^+^ and CD8^+^ T cells, and participate in the activation of NK cells [1]. T cell exosomel miRNAs are transferred into DCs in an antigen-specific manner [21]. In addition, it has been reported that regulatory T cell-released exosomes act as a suppressor against pathogenic Th1 responses in a miRNA-dependent manner [22]. These findings seem to indicate the inheriting donor cell functions in the released exosomes partly via miRNAs. CD8^+^ T cells play as a central effector in surveillance against malignancy [23, 24], suggesting the presence of cytotoxic or down-regulatory miRNAs against tumors in CD8-dominant T cell-derived exosomes used in this study. Indeed, some antitumor miRNAs reported previously were found in T cell-released exosome-dominant miRNAs by PubMed search (S1 Table) [25–36], having a possibility of T cell-derived exosomes as a tumoricidal effector, while further studies are necessary to clarify this supposition.

The difference of miRNA distribution pattern in the donor cells with the released exosomes (Fig 2, [21]), implying one possibility that RBP-mediated translocation of cellular miRNAs into exosome cargos occurs by G-recognition when multivesicular endosomes are generated in the donor cells. An *in silico* analysis using an RBPDB database to predict RBPs specific to 335 human T cell-released exosome-dominant miRNAs, compared to 75 miRNAs from donor T cell-released exosomes, revealed that FUS, SFRS13A and RBM4, in addition to RNPA2B1 as previously reported [16], were identified as strong candidate RBPs for the selective translocation of cellular miRNAs into exosome cargos (S2 Table). With the exception of FUS at 3.9%, these candidates were not predicted as donor T cell-dominant exosome miRNA-specific RBPs. In particular, SFRS13A could possibly be a central player in the concentration of exosomal miRNAs because of the wide recognition of G-rich domains. Further analysis by silencing the expression of these candidate RBPs will be necessary to clarify the relationship between G-rich exosome-dominant miRNAs and RBPs, including examination of RBP modifications, such as sumoylation or ubiquitination [16, 37].

A low significant positive correlation of G in sequences from HCT116-released exosome-dominant miRNAs was calculated by statistical analysis using Pearson’s correlation test, although a strong significance was indicated in other cases. Outliers positioned at a high X- and low Y-axis area or a low X- and high Y-axis area have been shown to affect positive correlation calculations using Pearson’s test [38]. In the current study, such biased miRNAs (e.g. human miR-1260b, -4286, -5100, -7975, and -7977) were scattered in an area of high exosomal miRNA value and low G percentage in HCT116-released exosome-dominant miRNAs, compared with A549- and K562-exosomal miRNAs, and therefore were determined to be the cause of the low significant positive correlation values. Indeed, HCT116 exosome-dominant miRNAs excluding 5 biased miRNAs resulted in the strong significant positive correlation of G (r = 0.123, P < 0.001). It is suggested that such outliers (miR-1260b, -4286, -5100, -7975, and -7977) are incorporated into exosome cargos from the donor cytoplasm by natural inflow rather than RBP-mediated active translocation because they exist at high levels in HCT116 cells.

Our selected GGAG sequence in exosome-dominant miRNA sequences is identical to one of four of nucleotide motifs reported previously [16]. However the other three motifs, UGAG, CCCU and UCCU, were not identified in our analysis, possibly due to changes in exosomal miRNA patterns by the activation state of the donor human T cells, such as different stimulation conditions of human T cells; the present study used a CD3-specific monoclonal antibody and RetroNectin, versus phorbol 12-myristate 13-acetate and ionomycin used in the previously published study [16]. The differences could also be due to inherent differences between the “*Toray 3D-gene*” and “*Agilent*” microarray chips for the detection of miRNA profiles. Fluctuations in miRNA patterns by the activation state of human T cells was negligible because the G-rich sequences, including a GGAG motif, were generally present in miRNAs from human tumor-, murine tumor- and murine T cell-released exosomes in addition to the exosomal miRNA sequences of human activated T cells in this study. Therefore, differences in the miRNA microarray chips could be crucial to the analysis of miRNA sequences. Although both Toray 3D-gene and Agilent microarray chips are extremely accurate and reproducible compared to quantitative RT-PCR [39], it seems that the probes on the microchips from Toray or Agilent are designed for detecting total miRNAs including pri, pre and mature forms, or mature miRNAs, respectively [40]. Mature miRNAs are stabilized by 3’-end adenylation or uridylation, and 3’-end uridylated miRNAs have been reported to be concentrated in exosomes [41]. Two CCCU and UCCU motifs may be identified only when using an agilent microarray chip for mature miRNAs.

In summary, the G-rich feature of exosomal miRNAs reveal important insights into the utilization of T cell-released exosomes for the treatment of cancer, and the development of nanomedicines by artificially introducing cytotoxic short RNAs, including miRNA and siRNA, into the exosome cargo in combination with a rule of 3’-end uridylation. The invention of comprehensive G-rich sequences and RBP-bound G-rich domains of exosomal miRNAs will revolutionize the introduction of siRNAs generally designed in a restricted G/C content into exosomes.

## Supporting Information

S1 Fig Surface molecules and mean diameter of human tumor cell-released exosomes. K562-, A549-, or HCT116-released exosomes bound with latex beads were treated with each indicated human surface molecule-specific mAb, and subjected to analysis by flow cytometry. The mean diameter of K562-, A549-, or HCT116-released exosomes was examined by NTA.
(TIF)

S2 Fig Surface molecules and mean diameter of murine cell-released exosomes. RAW264.7-, DUC18 CTL-, or BALB CD8^+^ T cell-released exosomes bound with latex beads were treated with each indicated murine surface molecule-specific mAb, and subjected to analysis by flow cytometry. The mean diameter of RAW264.7-, DUC18 CTL-, or BALB CD8^+^ T cell-released exosomes was examined by NTA.
(TIF)

S3 Fig Confirmation of the quantitative correlation of miRNAs between normalized raw data of microarray and RT-qPCR. Indicated 6 human T cell-released exosome-dominant or 3 donor T cell-dominant miRNAs were selected from the high E value and the high E/C value groups, or the high C value group by comparing the normalized raw data of microarray, respectively. RT-qPCR was performed by using primer-specific for the selected each miRNA. Data were expressed as the mean ± SD (duplicate) of the relative quantification of each miRNA.
(TIF)

S4 Fig Concentrated G in murine CTL-, human lymphoma- and murine macrophage-released exosome-dominant miRNA sequences. The indicated G patterns (no color in other bases and patterns) in miRNA sequences were visualized as a red color, and lined up in order from the largest amount of exosomal miRNA.
(TIF)

S5 Fig Concentrated G in miRNA sequences of A549- or HCT116-released exosomes. Percentage of G, maximal continuity of G, number of continuous G and maximum G-G interval in 1023 HCT116 or 619 A549 miRNA sequences were lined up in order from the highest ratio of exosome/donor T cell miRNAs. Pearson’s correlation test was performed, and the correlation coefficient (r) and p-value were calculated to confirm statistical significance of each G feature of exosome-dominant miRNA sequences.
(TIF)

S6 Fig Statistical anaylsis of each 4 base in CMS5a tumor-bearing BALB/c splenocyte-released exosome-dominant miRNA sequences. (A) The percentage of each base in cultured CMS5a-bearing BALB/c T cell-released exosomal miRNAs was indicated by different colors, and lined up in order from the highest value of exosomal miRNAs. (B) Pearson’s correlation test was performed to confirm statistical significance of the G-rich feature of CMS5a-bearing BALB/c T cell-released exosomal miRNA sequences. The correlation coefficient (r) and p-value were calculated between the percentage of each base (U, C, A or G) and exosomal miRNA values.
(TIF)

S1 Table Reported tumoricidal miRNAs in exosoma-dominant miRNAs. Tumoricidal miRNAs were selected from 335 exosome-dominant miRNAs by PubMed search.
(DOCX)

S2 Table Predicted RBPs specific for exosome-dominant miRNAs or donor human T cell-dominant miRNAs. Exosome-dominant miRNA-specific RBPs were predicted by using RBPDB database.
(DOCX)
