# Supplementary figures and images for: Guanine-Rich Sequences Are a Dominant Feature of Exosomal microRNAs across the Mammalian Species and Cell Types
Source: PLoS One. 2016 Apr 21;11(4):e0154134. doi: 10.1371/journal.pone.0154134 (PMC4839687; doi:10.1371/journal.pone.0154134)

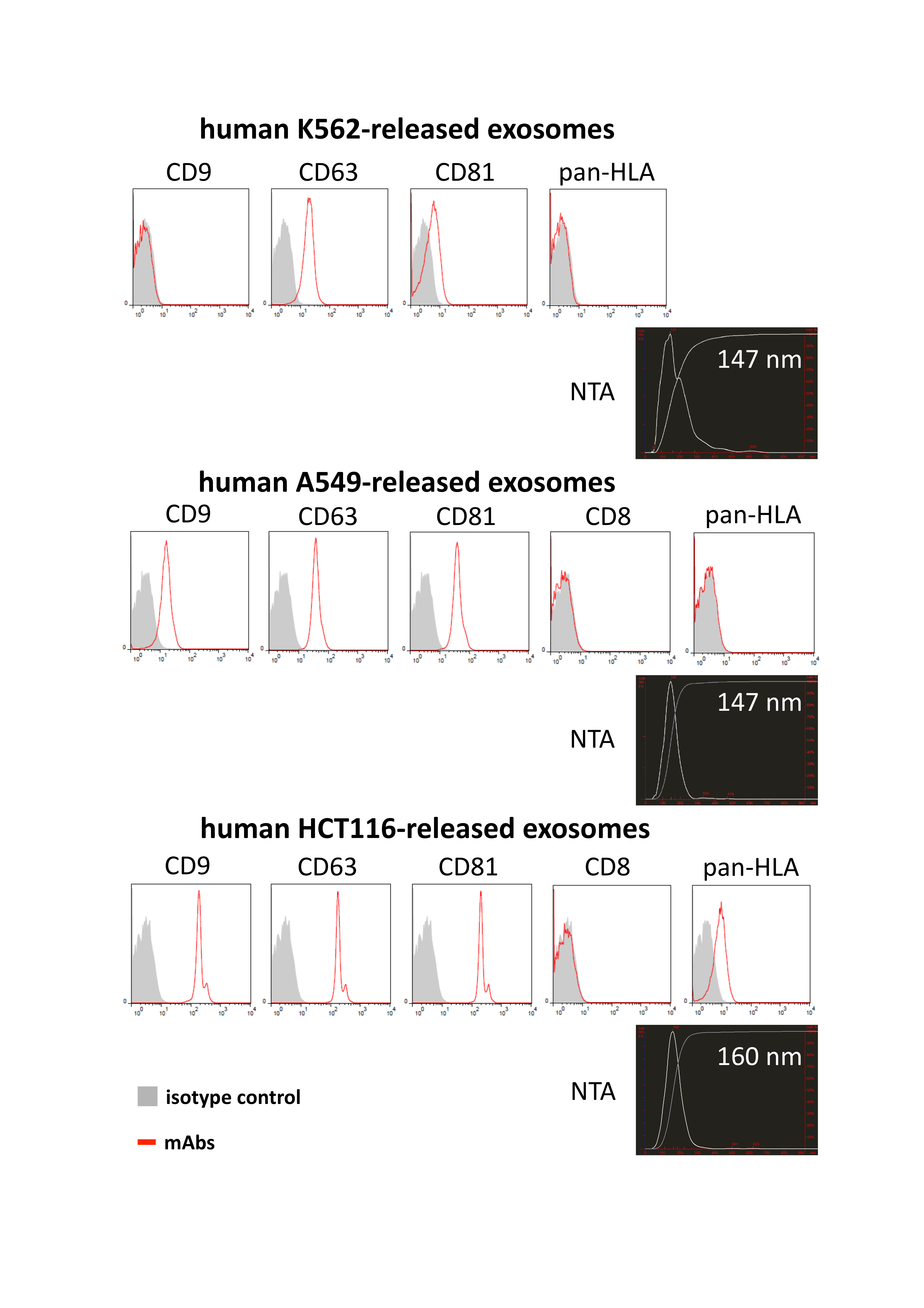

Supplement: S1 Fig — K562-, A549-, or HCT116-released exosomes bound with latex beads were treated with each indicated human surface molecule-specific mAb, and subjected to analysis by flow cytometry. The mean diameter of K562-, A549-, or HCT116-released exosomes was examined by NTA. (TIF) [file pone.0154134.s001.tif]

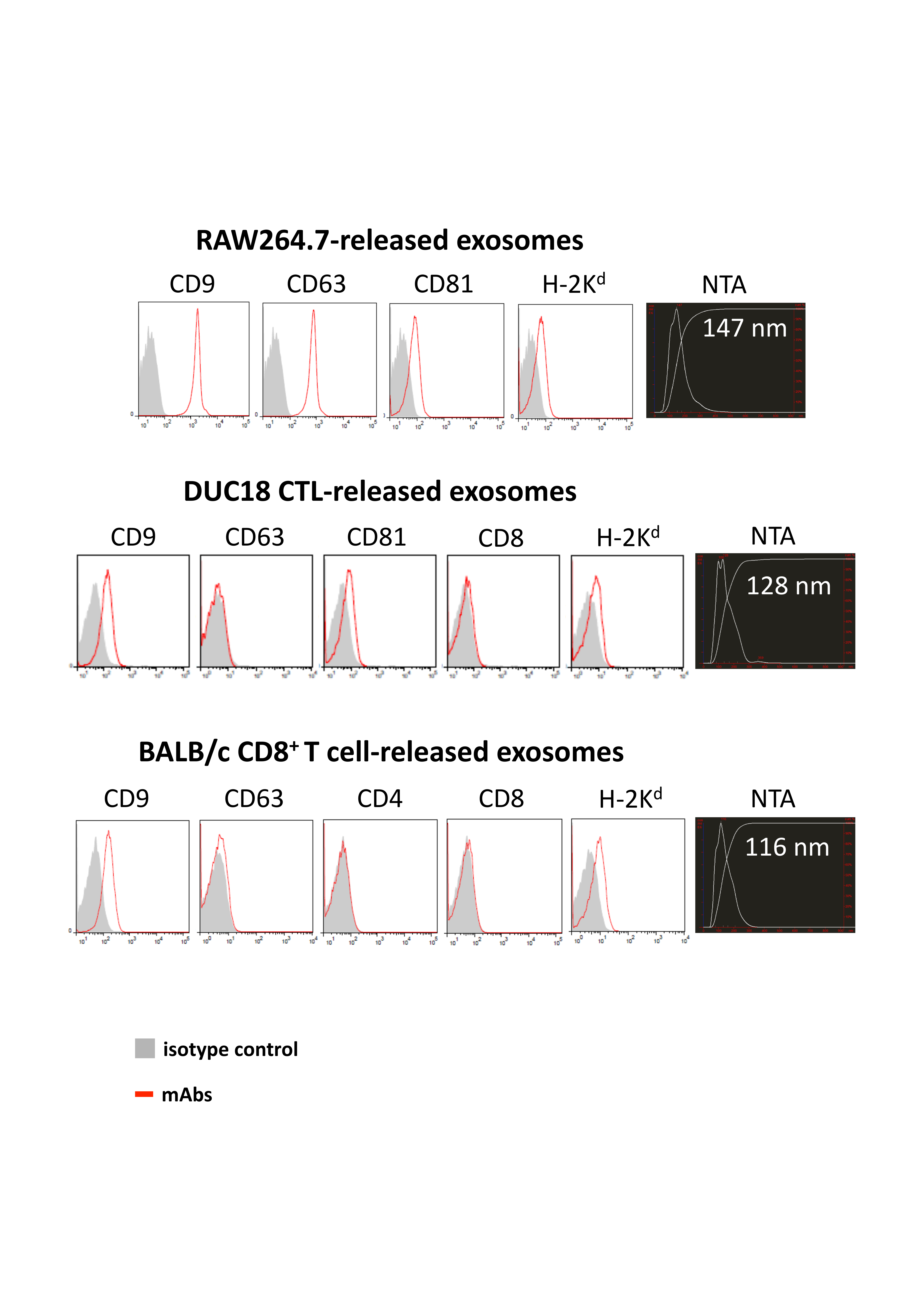

Supplement: S2 Fig — RAW264.7-, DUC18 CTL-, or BALB CD8+ T cell-released exosomes bound with latex beads were treated with each indicated murine surface molecule-specific mAb, and subjected to analysis by flow cytometry. The mean diameter of RAW264.7-, DUC18 CTL-, or BALB CD8+ T cell-released exosomes was examined by NTA. (TIF) [file pone.0154134.s002.tif]

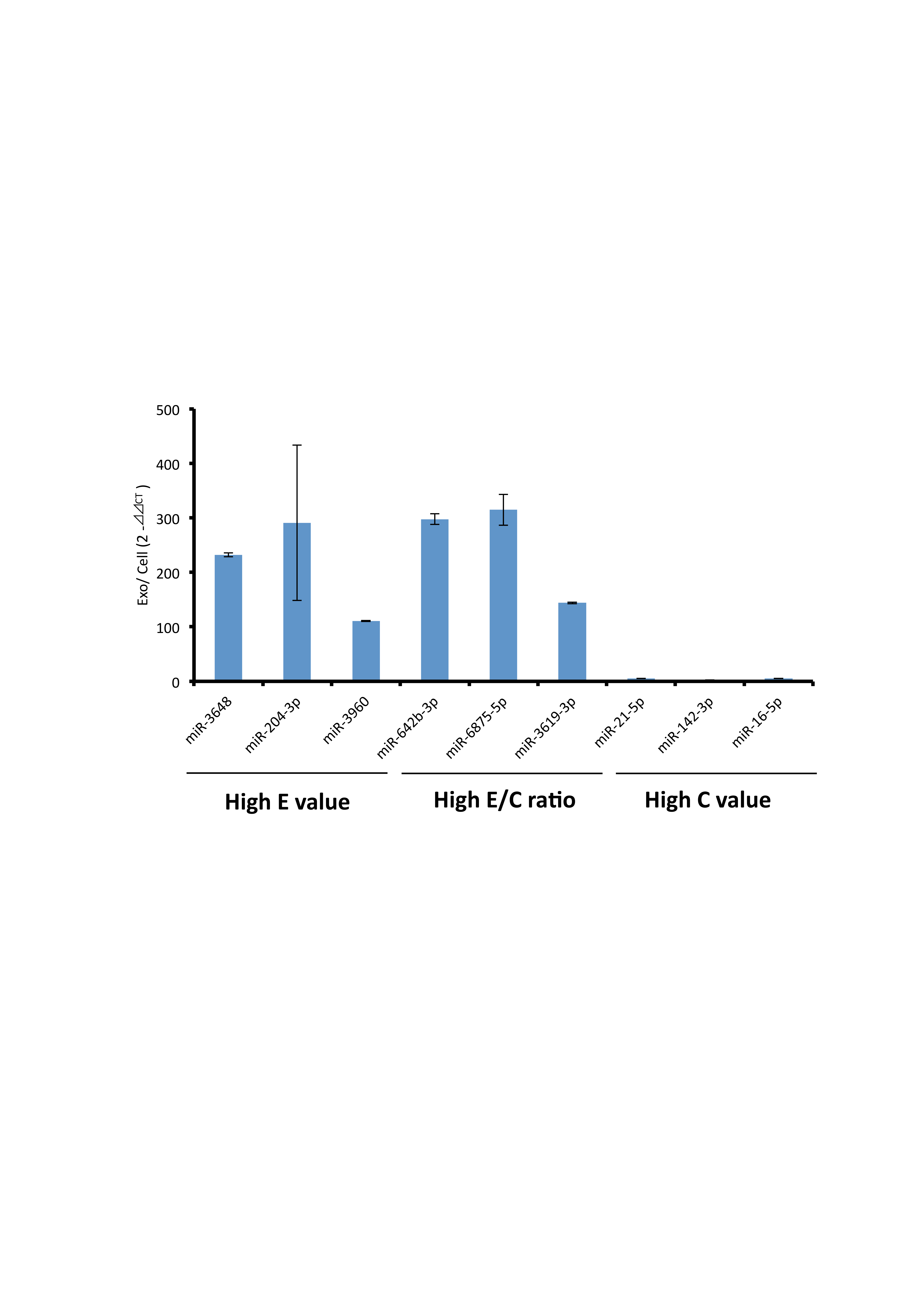

Supplement: S3 Fig — Indicated 6 human T cell-released exosome-dominant or 3 donor T cell-dominant miRNAs were selected from the high E value and the high E/C value groups, or the high C value group by comparing the normalized raw data of microarray, respectively. RT-qPCR was performed by using primer-specific for the selected each miRNA. Data were expressed as the mean ± SD (duplicate) of the relative quantification of each miRNA. (TIF) [file pone.0154134.s003.tif]

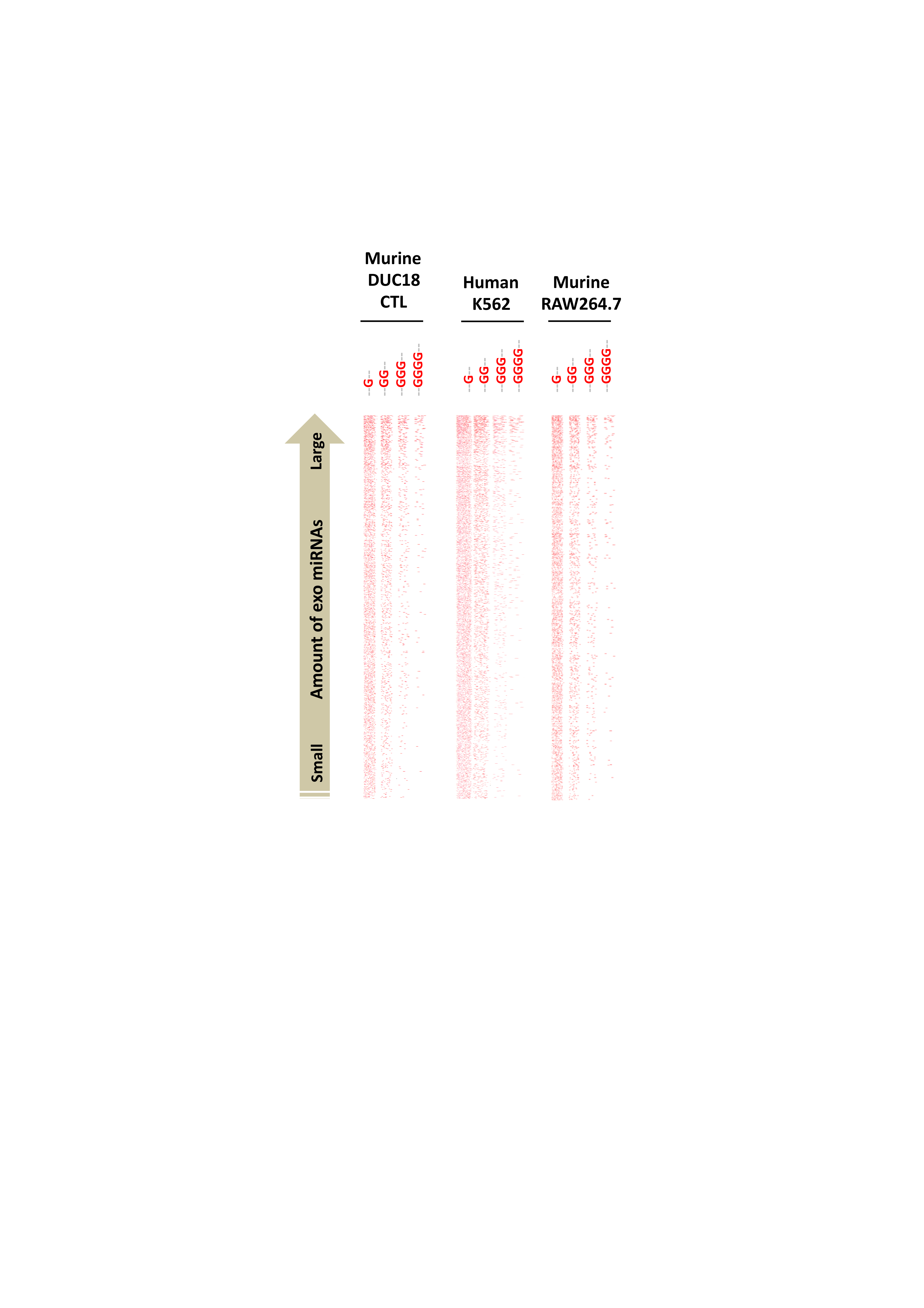

Supplement: S4 Fig — The indicated G patterns (no color in other bases and patterns) in miRNA sequences were visualized as a red color, and lined up in order from the largest amount of exosomal miRNA. (TIF) [file pone.0154134.s004.tif]

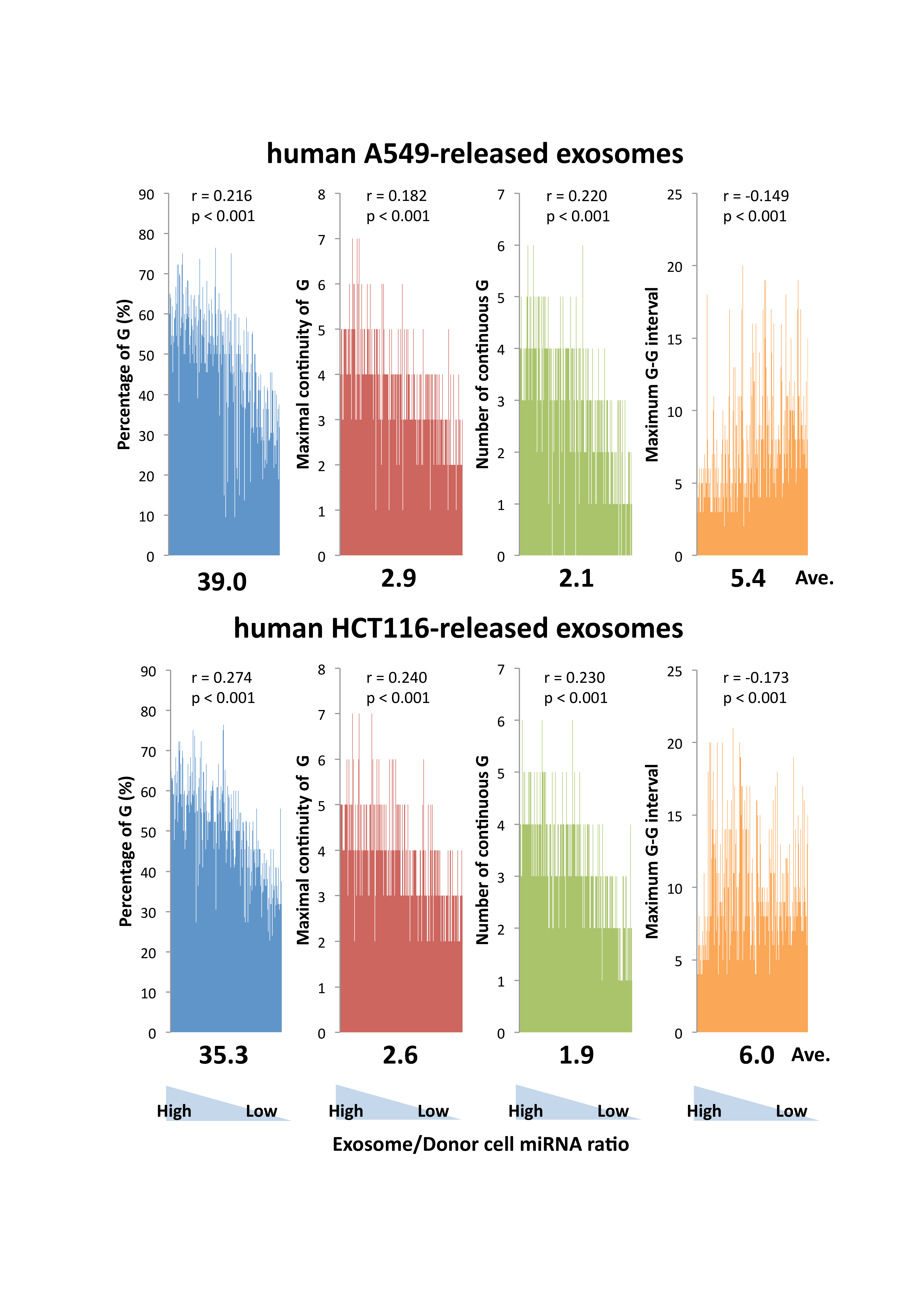

Supplement: S5 Fig — Percentage of G, maximal continuity of G, number of continuous G and maximum G-G interval in 1023 HCT116 or 619 A549 miRNA sequences were lined up in order from the highest ratio of exosome/donor T cell miRNAs. Pearson’s correlation test was performed, and the correlation coefficient (r) and p-value were calculated to confirm statistical significance of each G feature of exosome-dominant miRNA sequences. (TIF) [file pone.0154134.s005.tif]

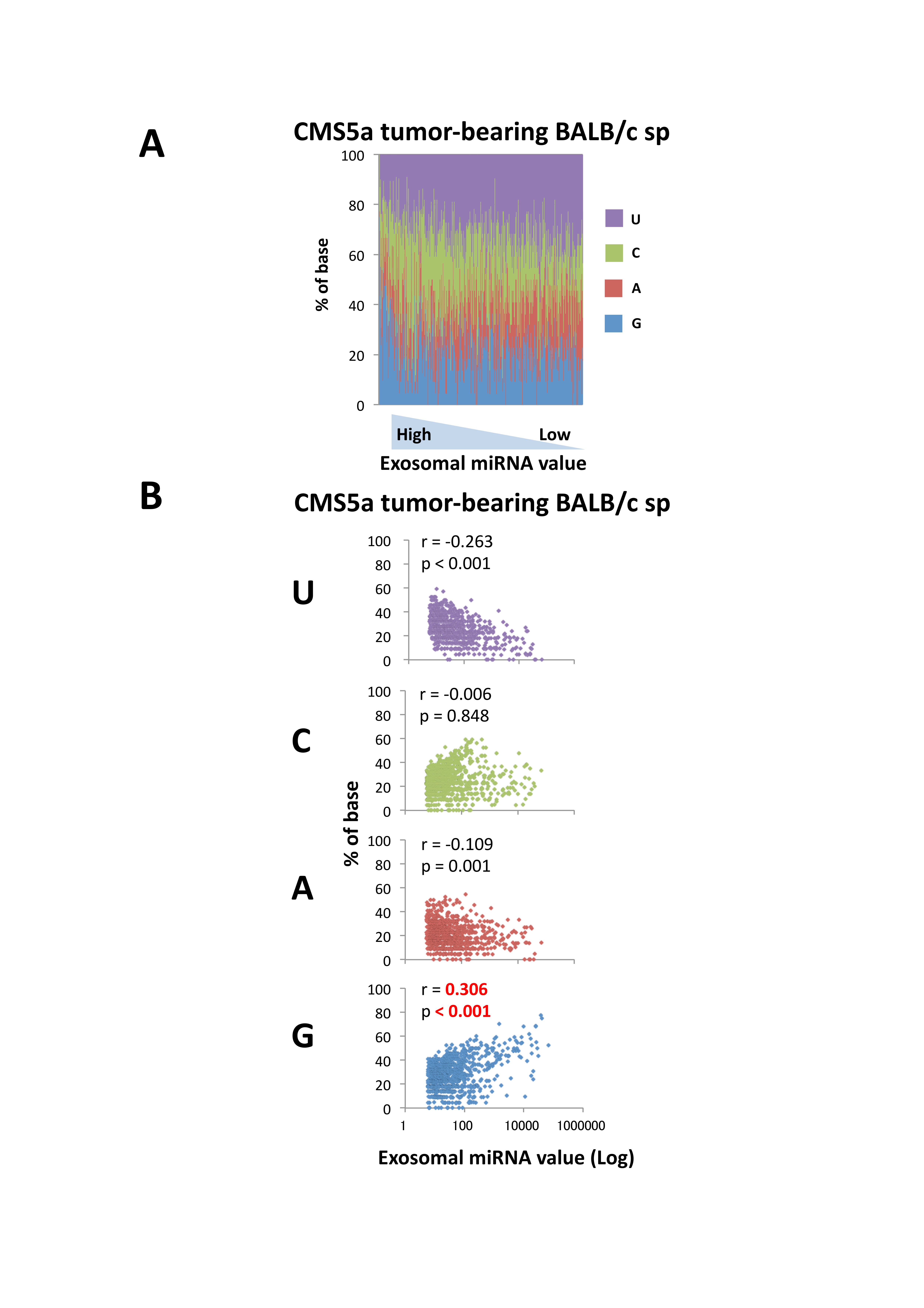

Supplement: S6 Fig — (A) The percentage of each base in cultured CMS5a-bearing BALB/c T cell-released exosomal miRNAs was indicated by different colors, and lined up in order from the highest value of exosomal miRNAs. (B) Pearson’s correlation test was performed to confirm statistical significance of the G-rich feature of CMS5a-bearing BALB/c T cell-released exosomal miRNA sequences. The correlation coefficient (r) and p-value were calculated between the percentage of each base (U, C, A or G) and exosomal miRNA values. (TIF) [file pone.0154134.s006.tif]
