# Supplementary material for: Guanine-Rich Sequences Are a Dominant Feature of Exosomal microRNAs across the Mammalian Species and Cell Types
Source: PLoS One. 2016 Apr 21;11(4):e0154134. doi: 10.1371/journal.pone.0154134 (PMC4839687; doi:10.1371/journal.pone.0154134)
Supplement: S1 Table — Tumoricidal miRNAs were selected from 335 exosome-dominant miRNAs by PubMed search. (DOCX) [file pone.0154134.s007.docx]

| miRNA | Anti-tumor functions | Ref. |
| --- | --- | --- |
| miR-204-3p | Growth inhibition of hepatocellular carcinoma endothelial cells | 25 |
| miR-296-5p | Prostate cancer suppressor by targeting Pin1 | 26 |
| miR-423-5p | Gastric cancer regulation by targeting TTF1 | 27 |
| miR-491-5p | Suppressor in ovarian, Era^+^ breast, and cervical cancers | 28-30 |
| miR-638 | Suppression of gastric cancer growth | 31 |
| miR-663a | Glioblastoma suppressor by targeting PIK3CD  Inhibition of pancreatic cancer progression | 32, 33 |
| miR-675-5p | Progression of NSCLC by downregulation | 34 |
| miR-718 | Inhibition of ovarian cancer progression | 35 |
| miR-765 | Suppression of prostate cancer progression | 36 |
