# Supplementary material for: Guanine-Rich Sequences Are a Dominant Feature of Exosomal microRNAs across the Mammalian Species and Cell Types
Source: PLoS One. 2016 Apr 21;11(4):e0154134. doi: 10.1371/journal.pone.0154134 (PMC4839687; doi:10.1371/journal.pone.0154134)
Supplement: S2 Table — Exosome-dominant miRNA-specific RBPs were predicted by using RBPDB database. (DOCX) [file pone.0154134.s008.docx]

| RBPs | Typical binding sites | Exo miRs (335) | Donor T miRs (76) |
| --- | --- | --- | --- |
| SFRS1 | AGGA UGGA | 101 (30.1%) | 11 (14.5%) |
| FUS | GGUG CGCG | 100 (29.8%) | 3 (3.9%) |
| EIF4B | GGAA GGAC | 91 (27.2%) | 11 (14.5%) |
| RBMX | CCAC CCAG | 84 (25.1%) | 28 (36.8%) |
| SFRS9 | AGGAG AGCAC | 55 (16.4%) | 11 (14.5%) |
| NONO | AGGGA | 38 (11.3%) | 6 (7.9%) |
| SFRS13A | AGAGGAG AGAGGGU | 31 (9.6%) | 0 (0%) |
| RBM4 | CGCG GCGCG | 25 (7.5%) | 0 (0%) |
| ELAVL1 | GUUU AUUU | 16 (4.8%) | 13 (17.1%) |
| MBNL1 | UGCU | 12 (3.6%) | 11 (14.5%) |
| KHSRP | GUCC | 9 (2.7%) | 0 (0%) |
| RNPA2B1 | UAGGGA | 7 (2.1%) | 0 (0%) |
